# Exploring Loneliness in Clinical and Sub‐Clinical Eating Disorders: A Systematic Review

**DOI:** 10.1002/erv.70023

**Published:** 2025-08-06

**Authors:** Elisa Rabarbari, Chiara Rossi, Fabio Frisone, Giuseppe Riva

**Affiliations:** ^1^ Department of Psychology Catholic University of the Sacred Heart Milan Italy; ^2^ Humane Technology Laboratory Catholic University of the Sacred Heart Milan Italy; ^3^ Applied Technology for Neuro‐Psychology Laboratory IRCCS Istituto Auxologico Italiano Via Magnasco 2 Milan Italy

**Keywords:** anorexia nervosa, binge‐eating disorder, bulimia nervosa, eating disorders, loneliness

## Abstract

**Objective:**

This review systematically examines the role of loneliness in clinical and subclinical Eating Disorders (ED), assessing its impact on symptom severity and exploring underlying mechanisms across different populations.

**Method:**

Following PRISMA guidelines and registered with PROSPERO (CRD42024565108), a comprehensive search was conducted in PubMed, Scopus, PsycINFO, and Web of Science. Inclusion criteria required quantitative measures of loneliness and ED symptoms in clinically diagnosed or subclinical populations. Pandemic‐related loneliness studies were excluded. Bias was assessed with the Downs and Black checklist.

**Results:**

A total of 15 studies with 2907 participants met the inclusion criteria. Findings revealed loneliness as a transdiagnostic factor in ED pathology. In clinical ED populations, loneliness was associated with increased symptom severity, particularly for Bulimia Nervosa (BN) and Binge Eating Disorder (BED), where emotional dysregulation was mediated by loneliness. Findings from subclinical samples suggest that loneliness may be an early psychosocial correlate of ED symptoms, particularly among young adults.

**Conclusion:**

Loneliness significantly contributes to ED development and persistence across clinical and subclinical populations, with potential implications for treatment. Social support and emotional regulation interventions may mitigate loneliness and improve ED outcomes. Future research should address gender differences and incorporate diverse populations to deepen understanding of this relationship.

**Trial Registration:**

PROSPERO Register: CRD42024565108.

## Highlights

- Loneliness is a transdiagnostic factor in ED, linked to increased symptom severity across clinical diagnoses, particularly in BN and BED.
- In subclinical populations, loneliness emerges as a salient psychosocial correlate of disordered eating behaviours, particularly in young adults, highlighting its relevance for early identification and preventive interventions.
- Loneliness mediates the relationship between emotional dysregulation and ED severity, indicating its critical role in maintaining maladaptive eating patterns.
- Emerging evidence suggests loneliness contributes to physiological dysregulation in ED patients, including increased inflammatory markers, emphasising the need for integrated biopsychosocial interventions.

## 1 Introduction

The relationship between loneliness and eating disorders is an emerging research field. Loneliness may contribute to the development and maintenance of disordered eating and eating behaviours through various psychological and social mechanisms (Levine 2012). For instance, feelings of isolation can lead to emotional dysregulation, where individuals may use food as a coping strategy to manage negative emotions, resulting in patterns like emotional eating or binge eating (Dingemans et al. 2017; Van den Eynde et al. 2011). Additionally, loneliness can exacerbate body dissatisfaction and low self‐esteem, which are well‐established risk factors for the onset of eating disorders (Culbert et al. 2015).

Loneliness is a pervasive and multifaceted human experience characterised by a subjective feeling of social isolation or a lack of meaningful connections with others (Peplau and Perlman 1982). Distinct from simply being alone, loneliness pertains to the perception of being disconnected, even when in the presence of others (Hawkley and Cacioppo 2010). The social needs model posits that humans have an innate need to form and maintain strong and stable interpersonal relationships, and failure to satisfy this need results in loneliness (Baumeister and Leary 2017). It arises from a perceived discrepancy between an individual's desired and actual levels of social relationships, implying a cognitive component that involves awareness of unmet social expectations (Peplau and Perlman 1982).

Recent theories underscore that loneliness is not merely a transient emotional state but involves a complex interplay of emotional, cognitive, and social factors with profound implications for psychological and physiological well‐being (Cacioppo and Cacioppo 2018). Moreover, irrational or distorted thinking plays a key role in loneliness by leading to deficits in sociability and social interactions, as a person's thoughts and beliefs about themselves and others directly impact their ability to form satisfying relationships (Peplau and Perlman 1982).

The importance of loneliness in mental health cannot be underestimated. It has emerged as a critical factor influencing the onset, maintenance, and exacerbation of various psychological disorders (Hawkley and Cacioppo 2010; Mann et al. 2022). Studies have consistently linked loneliness to a range of mental health issues, including depression, anxiety, substance use disorders, psychosis and even suicidal ideation (Cacioppo et al. 2006; Leigh‐Hunt et al. 2017; Rossi et al. 2023; Stickley and Koyanagi 2016). Loneliness acts as both a precursor and a consequence of mental health problems, creating a vicious cycle that can be challenging to break. Moreover, chronic loneliness has been associated with heightened stress responses, impaired immune functioning, and increased morbidity and mortality rates (Holt‐Lunstad et al. 2015). The global COVID‐19 pandemic has further amplified concerns about loneliness, as social distancing measures have inadvertently intensified feelings of isolation for many individuals (Loades et al. 2020).

Loneliness has been implicated as a contributing factor in the development and maintenance of Eating Disorders (ED), including Anorexia Nervosa (AN), Bulimia Nervosa (BN), and Binge‐Eating Disorder (BED), where maladaptive eating behaviours serve as coping mechanisms to manage feelings of social and emotional isolation (Levine 2012).

ED are severe mental health conditions and are characterised by dysfunctional eating behaviours and beliefs, distorted body image, and excessive concern with weight and shape (American Psychiatric Association 2022). In addition to the well‐established ED diagnoses, it is critical to acknowledge the inclusion of Eating Disorder Not Otherwise Specified (EDNOS) and Other Specified Feeding or Eating Disorders (OSFED) in the Diagnostic and Statistical Manual of Mental Disorders (DSM). EDNOS was first introduced in DSM‐IV (American Psychiatric Association 1994), while OSFED is included in DSM‐5 (American Psychiatric Association 2013) and DSM‐5‐TR (American Psychiatric Association 2022). OSFED describes individuals experiencing clinically significant distress and impairment in social, occupational, or other key function areas but who do not fully meet the criteria for AN, BN, or BED. These individuals often display symptoms typical of specific ED without meeting diagnostic thresholds, like the frequency of binge‐eating or purging or the weight criterion for AN. EDNOS, in contrast, applies when a clinician opts not to specify why criteria for a formal ED are not met or when information is limited. However, many individuals exhibit subclinical symptoms: significant disordered eating behaviours that do not meet the full diagnostic criteria for any ED (Stice et al. 2013) and they deserve attention as they can lead to substantial distress, impaired functioning, and are often precursors to fully developed disorders (Allen et al. 2013).

Individuals with ED symptoms may withdraw socially due to shame, stigma, or preoccupation with food and body image, increasing their feelings of loneliness (Treasure et al. 2015). This bidirectional relationship highlights the complex interplay between loneliness and disordered eating behaviours. Moreover, the use of social media, while potentially a source of connection, can sometimes exacerbate feelings of loneliness and contribute to unhealthy comparisons and body image concerns, further linking the two phenomena (Fardouly and Vartanian 2016).

Despite growing evidence on the prevalence of loneliness and its role in the development of ED, including subclinical symptoms, recent literature has yet to comprehensively explore this connection. While links between loneliness and disordered eating behaviours have been proposed, in‐depth analyses are limited. Thus, a systematic review of existing studies is needed to better understand the influence of loneliness on disordered eating and vice versa.

This review aims to examine and synthesise current evidence on the relationship between loneliness and both clinical and subclinical ED. By consolidating findings, we seek to clarify the extent to which loneliness may contribute to ED symptoms and how these behaviours may reinforce loneliness. Identifying literature gaps and potential mechanisms underlying this relationship can inform future research and clinical approaches, enhancing understanding and supporting targeted interventions.

## 2 Method

The current systematic review was pre‐registered in the PROSPERO register (CRD42024565108). It was carried out following the Preferred Reporting Items for Systematic Reviews and Meta‐Analyses (PRISMA).

### 2.1 Search Strategy

The literature process of the search was conducted in July 2024, through four databases: PubMed, Scopus, PsycINFO, and Web of Science.

The following string was used to filter the titles, abstracts, and keywords of the articles: loneliness AND ‘eating disorder*’ OR ‘disordered eating’ OR ‘eating symptoms” OR anorexia OR bulimia OR ‘binge eating’.

To offer a broad panoramic of the current state of the art on the topic, it has not been defining a beginning year of publication for the articles to be included. Screening was carried out by two reviewers (E.R. and C.R.). To minimise bias, a third independent judge (F.F) was included to evaluate articles in which the two main judges did not agree.

The complete list of the extracted articles was imported into EndNote (Clarivate 2023) to remove duplicates, and then it was imported into Rayyan (Ouzzani et al. 2016) to check the title and the abstract.

### 2.2 Inclusion and Exclusion Criteria

Studies were included based on the following criteria: (a) they were published in English; (b) they were quantitative research articles; (c) they included studies where participants completed validated self‐report measures about Loneliness and ED symptomatology, and/or (d) they met a current diagnosis of ED according to the Diagnostic and Statistical Manual of Mental Disorders, Third Edition (DSM‐III, American Psychiatric Association 1987), Fourth Edition (DSM‐IV, American Psychiatric Association 1994; DSM‐IV‐TR, American Psychiatric Association 2000) and Fifth Edition (DSM‐5, American Psychiatric Association 2013).

The initial intention was to include only studies with a formal diagnosis of ED. However, due to the limited availability of such research, the criteria were broadened to include both studies involving formally diagnosed ED and those with subclinical samples, characterised by clinically significant ED symptoms assessed through validated questionnaires. This decision is informed by the rationale that examining the role of loneliness in subclinical populations may provide valuable insights for early identification and prevention strategies (Crowe et al. 2024).

No restrictions have been placed on participants' gender and ethnicity.

Studies investigating loneliness during the COVID‐19 pandemic were excluded due to the challenge of differentiating between loneliness as a personal experience or as related to the social environment, and the loneliness stemming from the enforced social isolation during the global pandemic, which impacted the entire population. Thus, qualitative studies, longitudinal studies, reviews, thesis, conference proceedings, notes, letters to the editor, and research protocols were excluded. Systematic reviews were not included, but their references have been screened for the selection of relevant articles.

### 2.3 Risk of Bias Assessment

The risk of bias was assessed using the methodological quality checklist proposed by Downs and Black (1998) designed to evaluate both randomized and non‐randomized comparative studies. Their checklist includes criteria such as reporting strategy, external validity, internal validity, and power. Examples of the checklist items include: ‘Is the hypothesis/aim/objective of the study clearly described?’, ‘Are the statistical tests used to assess the main outcomes appropriate?’ and ‘Were the main outcome measures used accurate (valid and reliable)?’. Any disagreements or ambiguities were resolved through consensus. The results of the risk of bias assessment are detailed in Table 1.

**TABLE 1:** Risk of bias assessment using the Downs and Black Checklist (1998).

	Esplen et al. (2000)	Harney et al. (2014)	Makri et al. (2022)	McNamara et al. (2022)	Southward et al. (2014)	Meneguzzo, Terlizzi, et al. (2024)	Masheb and Grilo (2006)	Coric and Murstein (1993)	Marffy et al. (2024)	Chang et al. (2014)	Hussain (2021)	Pritchard and Yalch (2009)	Rotenberg et al. (2013)	Wright and Pritchard (2009)	Mason (2024)
Question 1	1	1	1	1	1	1	1	1	1	1	1	1	1	1	1
Question 2	1	1	1	1	1	1	1	1	1	1	1	1	1	1	1
Question 3	1	1	1	1	1	1	1	1	1	1	1	1	1	1	1
Question 4	1	1	1	1	1	1	1	1	1	1	1	1	1	1	1
Question 5	1	1	1	1	1	2	1	1	1	1	1	1	1	1	1
Question 6	1	1	1	1	1	1	1	1	1	1	1	1	1	1	1
Question 7	1	1	1	1	1	1	1	1	1	1	1	1	1	1	1
Question 8	0	0	0	0	0	0	0	0	0	0	0	0	0	0	0
Question 9	1	1	1	1	1	1	1	1	1	1	1	1	1	1	1
Question 10	1	1	1	1	1	1	1	1	1	1	1	1	1	1	1
Question 11	1	1	1	1	1	1	1	1	1	1	1	1	1	1	1
Question 12	1	1	1	1	1	1	1	1	1	1	1	1	1	1	1
Question 13	NA	NA	NA	NA	NA	NA	NA	NA	NA	NA	NA	NA	NA	NA	NA
Question 14	1	1	1	1	1	1	1	1	1	1	1	1	1	1	1
Question 15	1	1	1	1	1	1	1	1	1	1	1	1	1	1	1
Question 16	1	0	0	1	0	1	0	0	1	0	1	0	0	1	0
Question 17	NA	NA	NA	NA	NA	NA	NA	NA	NA	NA	NA	NA	NA	NA	NA
Question 18	1	1	1	1	1	1	1	1	1	1	1	1	1	1	1
Question 19	0	1	1	1	1	1	1	1	0	0	0	0	0	0	0
Question 20	1	1	1	1	1	1	1	1	1	1	1	1	1	1	1
Question 21	1	1	1	1	1	1	0	1	1	1	1	1	0	0	0
Question 22	1	1	1	1	1	1	1	1	1	1	1	1	1	1	1
Question 23	NA	NA	NA	NA	NA	NA	NA	NA	NA	NA	NA	NA	NA	NA	NA
Question 24	1	0	1	1	1	1	0	1	1	1	0	1	1	1	1
Question 25	1	0	1	1	1	1	0	1	1	1	0	1	1	1	1
Question 26	NA	NA	NA	NA	NA	NA	NA	NA	NA	NA	NA	NA	NA	NA	NA
Question 27	0	0	0	0	0	0	0	0	3	0	0	0	0	0	0
Tot	20	18	20	21	20	22	17	20	23	19	18	19	18	19	18

*Note:* 1. 0 = No, 1 = Yes, NA = Not Applicable. Only question 5 could be answered 0 = No, 1 = Partially, 2 = Yes. Cut‐off total score: 24–28 points (excellent), 19–23 points (good), 14–18 (fair), 14 points or less (poor).

## 3 Results

### 3.1 Data Extraction and Study Characteristics

**FIGURE 1:**
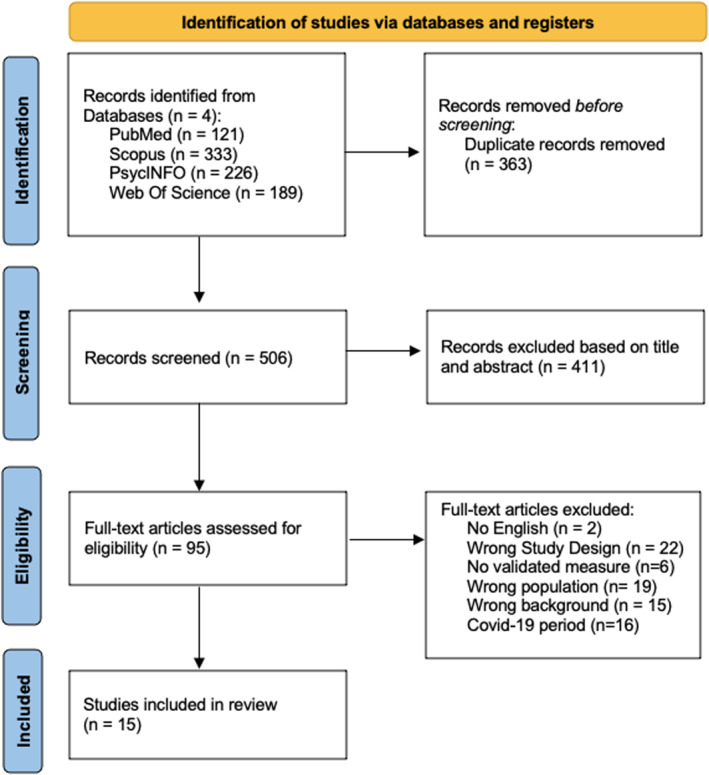
PRISMA flow diagram for study selection.

The PRISMA flow diagram for papers selection is shown in Figure 1.

The initial database search yielded 869 studies. Following the removal of duplicates (*n* = 363), 506 articles remained for abstract screening. After applying the inclusion criteria during abstract screening, a final total of 95 studies were deemed eligible for inclusion. Ultimately, after assessing the eligibility of full‐text articles, 15 studies were included in the final analysis.

The reviewers independently extracted the following data: authors of the study, country, study design, sample, measures used for ED and loneliness assessment, and the primary outcomes. Data are available in Table 2.

**TABLE 2:** Studies characteristics according to extraction parameters.

Authors, years	Country	Design	Sample	Measures	Main outcomes
Esplen et al. (2000)	Canada	Clinical study (part of randomized trial)	50 subjects (48 F, 2 M) CC: BN Age: *M* = 26.6, SD = 6	Eating disorder inventory (EDI) Eating attitudes Test‐26 (EAT‐26) Aloneness/Evocative memory scale	Individuals with BN who have difficulties with affect regulation (including self‐soothing and evocative memory) also experience higher levels of loneliness.
Harney et al. (2014)	USA	Cross‐sectional study	155 F subjects CC: ED (an, BN, EDNOS) Age: *M* = 21.78, SD = 4.28 Groups: Active ED (*N* = 53), partially recovered (*N* = 15), fully recovered (*N* = 20), and HC (*N* = 67) Ethnicity: 91.6% caucasian, 1.9% Asian, 1.3% African American, 5% biracial/Ethnic	Eating disorders longitudinal interval follow‐up evaluation (LIFE EAT II) Eating disorder examination‐questionnaire (EDE‐Q) UCLA loneliness scale (version 3)	Controls, fully recovered, and partially recovered individuals reported similar levels of loneliness that were significantly lower than those reported by the active eating disorder group.
Makri et al. (2022)	Greece	Case‐control study	61 subjects (57 F, 4 M) CC: ED (AN, BN, BED, atypical ED) Age: *M* = 33.3; SD = 11.7 Groups: ED (*N* = 32), HC (*N* = 29)	Eating disorder examination questionnaire (EDE‐Q) UCLA loneliness scale	ED patients showed higher levels of loneliness compared to HC and loneliness was predicted by social support satisfaction.
McNamara et al. (2022)	Ireland	Cross‐sectional study	82 subjects (70 F, 1 M, 1 transgender, 1 non‐binary, 9 not reported) CC: ED (AN, BN, AN/BN, EDNOS, multiple diagnosis, BED) Age: *M* = 28.95, SD = 8.33	Eating attitudes scale (EAT‐16) Three‐item loneliness scale	Loneliness was positively associated with ED symptom severity and served as a mediator between family identification and ED symptom severity.
Southward et al. (2014)	USA	Cross‐sectional study	107 F subjects CC: BN, BED Age: *M* = 35.2, SD = 12.5 Ethnicity: 69.2% caucasian, 12.1% African American, 12.1% hispanic, 2.8% Asian American, 3.7% other	Eating disorder Examination—14th edition (EDE) UCLA loneliness scale (version 3)	Loneliness significantly mediated the relationship between emotion dysregulation and BN/BED psychopathology.
Meneguzzo, Terlizzi, et al. (2024)	Italy	Cross‐sectional study	97 F subjects CC: ED (an, BN, EDNOS) Age: *M* = 23.7, SD = 7,74 Ethnicity: White	Eating disorder examination questionnaire (EDE‐Q) UCLA loneliness scale	Higher loneliness was correlated with greater severity in eating disorder psychopathology, particularly in dietary restraint and weight concerns.
Masheb and Grilo (2006)	USA	Cross‐sectional study	220 subjects (172 F, 48 M) CC: BED Age: *M* = 45.2, SD = 8.8 Ethnicity: 79.6% caucasian, 10.9% African American, 8.2% hispanic American, 1.4% other	Eating disorder examination‐questionnaire (EDE‐Q) Three‐factor eating questionnaire (TFEQ) Emotional overeating questionnaire (EOQ)	Loneliness is a significant emotional trigger for overeating and binge eating in individuals with BED.
Coric and Murstein (1993)	USA	Cross‐ sectional study	248 F subjects CC: BN Age: *M* = 19.6 Group: BN (*N* = 28), binge‐purge (*N* = 43); HC (*N* = 177)	Bulimia test (BULIT) UCLA loneliness scale	Significantly higher levels of loneliness were found in the BN group compared to both binge‐purge and HC.
Marffy et al. (2024)	UK	Cross‐sectional study	165 subjects (154 F, 3 M, 8 others) Age: *M* = 27.54 Ethnicity: 98.8% western countries (UK, Canada, USA), 1.2% Asia	Eating disorder examination questionnaire (EDE‐Q 6.0) UCLA loneliness scale (version 3)	Higher levels of loneliness were associated with significantly higher eating disorder severity.
Chang et al. (2014)	USA	Cross‐sectional study	301 F subjects Age: *M* = 19.71, SD = 1.31 Ethnicity: 70.4% european American, 12% Asian American, 3.7% African American, 2% hispanic, others	EDI: Drive for thinness, bulimia, body dissatisfaction Revised UCLA loneliness scale	Loneliness was found to be a significant predictor of eating disturbances and added significant incremental variance in predicting eating disturbances beyond what was accounted for by BIS/BAS motives, especially for bulimic symptoms.
Hussain (2021)	Pakistan	Correlation research design	210 subjects (137 F 73 M) Age: Approximately 21 Ethnicity: Pakistan	Eating disorder inventory (EDI) Three factor eating Questionnaire‐R‐18 UCLA loneliness scale (version 3)	The study found no significant relationship between loneliness and dysfunctional eating patterns.
Pritchard and Yalch (2009)	USA	Cross‐sectional study	245 subjects (147 F, 98 M) Age: *M* = 21.02, SD = 5.32 Ethnicity: 88% white, 6% hispanic, 2% African American, 1% Asian, 3% other	EDI: Drive for thinness, bulimic symptoms, and body dissatisfaction. UCLA loneliness scale	Loneliness mediated the relationship between interpersonal dependency and body dissatisfaction for both men and women and was significantly related to all three measures of disordered eating in men, but only to body dissatisfaction in women.
Rotenberg et al. (2013)	UK	Cross‐sectional study	137 subjects (81 F, 56 M) Age: *M* = 21.10; SD = 4.9	Stirling eating disorder scale (SEDS): Bulimic cognition and behaviour UCLA loneliness scale	Higher bulimic symptoms were associated with increased loneliness. The relation between bulimic symptoms and loneliness was mediated by lower trust in close relationships.
Wright and Pritchard (2009)	USA	Cross‐sectional study	273 subjects (120 F, 153 M)	Eating attitudes Test‐26 (EAT‐26) UCLA loneliness scale	Loneliness was significantly related to disordered eating behaviours and emerged as a significant predictor of disordered eating, even after accounting for the influence of mass media and gender.
Mason (2024)	USA	Cross‐sectional study	556 F subjects Age: *M* = 22.42, SD = 6.61 Ethnicity: 46.1% white, 34.7% black, 12.0% two or more races, 4.0% Asian, 1.4% American indian/Alaskan native, 0.9% native Hawaiian/Other pacific islander, 0.9% other. 7% hispanic, 93% non‐hispanic.	EDI: Bulimia scale (EDI‐B) Eating disorder diagnostic scale (EDDS) Friendship scale: Assessed loneliness	Loneliness was significantly associated with greater binge eating and moderated the relationship between affective symptoms and binge eating.

*Note:* 2: Participants' diagnoses were outlined following the Diagnostic and Statistical Manual of Mental Disorders (DSM‐III‐R, IV, IV‐TR or 5).

Abbreviations: AN = Anorexia; BED = Binge Eating Disorder; BN = Bulimia; CC = Clinical Condition; ED = Eating Disorders; EDNOS = Eating Disorder Not Otherwise Specified; HC = Healthy Control.

Overall, the included articles comprise a total of 2907 participants, aged 15 to 64. The female gender was the most represented (2450 women, 438 men, 19 non‐binary or other). Only four studies reported on samples with a meaningful proportion of men, and only one of these involved a clinical population.

Most of the studies were conducted in the United States (=8), two in the United Kingdom, one in Italy, one in Canada, one in Ireland, one in Greece, and one in Pakistan. Of the 15 included studies, 8 were classified as clinical studies, defined as those that utilised standardized clinical interviews for diagnosis and explicitly reported formal ED diagnosis in the articles. Among these clinical studies, 230 had BED, 207 had AN, 174 had BN, 24 had AN/BN, 67 had EDNOS and 4 had multiple diagnoses (Coric and Murstein 1993; Esplen et al. 2000; Harney et al. 2014; Makri et al. 2022; Masheb and Grilo 2006; McNamara et al. 2022; Meneguzzo, Terlizzi, et al. 2024; Southward et al. 2014). The remaining seven studies were classified as subclinical, characterised by the exclusive use of self‐report questionnaires, even when employing clinically significant cut‐off scores, without the presence of a formal ED diagnosis. Specifically, these studies examined undergraduate and college students exhibiting clinically significant symptoms of eating disorders (Chang et al. 2014; Hussain 2021; Marffy et al. 2024; Mason 2024; Pritchard and Yalch 2009; Rotenberg et al. 2013; Wright and Pritchard 2009).

This distinction between clinical and subclinical studies was established to enable a detailed and systematic analysis of the findings. However, individuals classified as subclinical based solely on self‐report measures might still meet formal diagnostic criteria for an ED. Self‐reported symptoms can reflect clinically relevant conditions, including OSFED, as defined in the DSM‐5‐TR. Nevertheless, due to the absence of a formal diagnostic assessment, they were not categorised within the clinical studies group. This methodological limitation should be considered when interpreting the results, particularly given the diagnostic complexity and heterogeneity of eating disorders.

All the studies have the common aim of exploring the role of loneliness in psychopathology ED. They all primarily used DSM‐III‐R, IV, IV‐TR, or 5 criteria (American Psychiatric Association 1987, 1994, 2000, 2013) and clinical interviews, based on them, for diagnosing AN, BN, BED, and/or EDNOS. For assessing Eating Disorders symptoms various questionnaires were used, including Eating Disorder Examination‐Questionnaire (EDE‐Q, Fairburn et al. 1993; EDE‐Q 6.0, Fairburn and Beglin 2008), Eating Disorder Inventory (EDI, Garner et al. 1983), Eating Attitudes Test‐26 (EAT‐26; Garner et al. 1982), Three‐Factor Eating Questionnaire (TFEQ‐R‐18, Karlsson et al. 2000; TFEQ, Stunkard and Messick 1985), Bulimia Test (BULIT, Smith and Thelen 1984); Stirling Eating Disorder Scale (SEDS, Williams et al. 1994), Eating Disorder Diagnostic Scale (EDDS, Stice et al. 2000); Eating Disorders Longitudinal Interval Follow‐up Evaluation (LIFE EAT II; Herzog et al. 1993). Instead, loneliness was mainly assessed with the UCLA Loneliness Scale (Russell et al. 1980; Version 3, Russell 1996), a widely validated and comprehensive measure of subjective feelings of social isolation and dissatisfaction with social relationships. In one study, a modified version of the UCLA was used, Aloneness/Evocative Memory Scale (Esplen et al. 2000), which includes additional items capturing the inability to evoke comforting relational memories and more extreme experiences of aloneness. Other studies employed more abbreviated or focused instruments, such as the Three‐item loneliness scale (Hughes et al. 2004), which captures general loneliness with fewer items, and the Friendship Scale (Hawthorne 2006), which combines elements of social connectedness and loneliness. Finally, one study used the Emotional Overeating Questionnaire that measures the frequency of overeating in response to six emotions, including loneliness (EOQ, Arnow et al. 1995).

The studies include psychological assessments and correlational analyses. To systematise results and better understand the impact of loneliness in ED, we pooled the results according to two categories: (1) clinical sample and (2) subclinical sample.

In the following paragraphs, studies characteristics and results are reported.

### 3.2 Clinical Sample

Throughout eight clinical studies on loneliness in patients with ED, consistent patterns were observed, highlighting the important role of loneliness in both the development and maintenance of ED symptoms. These results provide convincing evidence that loneliness exacerbates emotional distress and affects regulation, which contributes to the severity of ED, especially BN and BED.

Makri and colleagues (2022) found that individuals with ED experienced significantly higher levels of loneliness than healthy controls (*p* < 0.001). Patients with ED scored an average of 51.03 on the UCLA Loneliness Scale, compared to 34.66 in the healthy control group, indicating a substantial gap in subjective feelings of loneliness and isolation between the two groups. Notably, loneliness levels remained consistent across different ED diagnoses, highlighting it as a transdiagnostic factor. The significant association between loneliness and reduced social connectedness suggests a role for loneliness in exacerbating ED symptoms across various diagnoses. The study also found that loneliness was significantly correlated with social support satisfaction (negatively) and depressive symptomatology (positively) in ED patients. Still, just the degree of satisfaction with social support was found to be the statistically significant predictor of loneliness levels in ED patients.

Coric and Murstein (1993) study underscores the distinct role of loneliness in differentiating individuals with BN from those exhibiting subclinical binge‐purge behaviours and controls. In their sample of female college students, 11.3% met the strict DSM‐III‐R diagnostic criteria for BN, 17.3% displayed subclinical binge‐purge behaviours, and 71.4% constituted the control group without significant eating pathology. The findings revealed a robust association between loneliness and BN, with individuals in the BN group reporting significantly higher levels of loneliness compared to both the subclinical and control groups. Furthermore, loneliness in the BN group was strongly correlated with greater depressive symptomatology and reduced self‐esteem, indicating a complex interplay of emotional factors. These results suggest that loneliness may serve as a critical psychological component in BN, exacerbating emotional distress and potentially contributing to the maintenance of the disorder.

Along this line, Harney et al. (2014) demonstrated that loneliness levels were elevated in individuals with active ED yet normalised among those who were partially or fully recovered, as well as control participants. This indicates that the reduction of loneliness could be associated with recovery progression in ED patients, suggesting the potential benefit of integrating interventions targeting social connectedness and loneliness in ED treatment approaches.

Esplen and colleagues (2000) provide valuable insights into the role of loneliness in BN introducing the concept of ‘aloneness’ to describe an overwhelming sense of isolation, emotional emptiness, and an inability to derive comfort from memories or social interactions. Their findings indicate significant negative correlations between loneliness and self‐soothing capacities (soothing receptivity), as well as positive correlations between loneliness and impaired evocative memory. These results suggest that loneliness in individuals with BN extends beyond social deficits, reflecting deeper disruptions in psychological and interpersonal functioning. The inability to draw comfort from relational or internal resources may contribute to the maintenance of eating disorder pathology by exacerbating emotional distress and impairing adaptive coping mechanisms.

In a study examining emotional regulation, Southward et al. (2014), found that loneliness significantly mediated the relationship between emotion dysregulation and ED severity in women with BN and BED. Individuals who struggled with emotional regulation reported heightened loneliness, which in turn was linked to increased ED severity. This suggests that addressing loneliness in interventions could be crucial for improving emotional regulation and reducing ED symptoms.

Similar to the previous study, Masheb and Grilo’s (2006) findings further supported the link between loneliness and ED severity in BED, showing that loneliness‐induced emotional eating was strongly correlated with binge‐eating frequency and concerns about weight and body shape. Notably, women reported higher emotional overeating in response to loneliness than men, suggesting a gender‐specific response pattern that may warrant tailored interventions targeting emotional triggers in ED management.

Moreover, McNamara and colleagues' study (2022) extended the discussion on loneliness, highlighting its mediating role between family identification and ED symptom severity, where stronger family identification reduced loneliness, thereby alleviating ED symptoms. These findings underscore the importance of family and social support as protective factors in mitigating loneliness among individuals with ED.

Finally, the group of Meneguzzo, Terlizzi, et al. (2024) added a novel perspective by examining the biological impact of loneliness in ED patients. Higher loneliness levels were associated with increased childhood physical neglect and elevated inflammatory markers, linking loneliness to both psychological distress and physiological dysregulation. The data suggest that loneliness, beyond its emotional impact, may have significant physical consequences, highlighting its relevance as a complex psychosocial correlate in the context of ED pathology.

Together, these studies collectively underscore loneliness as a multidimensional construct affecting ED patients across emotional, psychological, and biological dimensions.

### 3.3 Sub‐Clinical Sample

This category encompasses studies focused on investigating the presence of loneliness and its correlation with symptoms of ED, considering undergraduate and college student populations.

Wright and Pritchard’s study (2009) explored how loneliness, media influence, and gender together contribute to disordered eating. While mass media influence had the strongest effect, loneliness still accounted for a smaller but significant portion of the variance in eating behaviours, particularly in young women. Loneliness was significantly related to disordered eating behaviours, suggesting that low levels of peer attachment and social support may contribute to disordered eating patterns.

Rotenberg and colleagues (2013) focused on bulimic symptoms and their relationship to psychosocial factors, specifically trust in close relationships and the willingness to disclose personal information. They found that individuals with higher bulimic symptoms showed lower levels of trust, leading to reduced self‐disclosure and, consequently, increased loneliness. Their structural model indicated that bulimic symptoms indirectly contributed to loneliness, with trust and disclosure acting as mediators. This suggests that loneliness in individuals with bulimic symptoms may stem from complex interpersonal dynamics beyond simple social isolation.

Chang and colleagues (2014) examined the relationship between eating disturbances, including drive for thinness, bulimic symptoms, and body dissatisfaction, and loneliness, while also considering the role of the Behavioural Inhibition System (BIS), linked to avoidance behaviours, and the Behavioural Activation System (BAS), associated with approach behaviours. The results indicated that loneliness was positively correlated with all three types of eating symptoms. Specifically, women experiencing loneliness were more likely to exhibit symptoms of ED, with loneliness uniquely predicting additional variance in eating disturbances beyond what was explained by BIS/BAS motives. Interestingly, loneliness showed a stronger association with bulimic symptoms compared to drive for thinness or body dissatisfaction, potentially due to the solitary nature of bingeing and purging behaviours. The findings highlighted loneliness as a critical factor in the maintenance and exacerbation of bulimic symptoms, emphasising the importance of social context in understanding and treating ED.

Similarly, Pritchard and Yalch (2009) analysed the relationship between ED symptoms ‐ such as the drive for thinness, bulimic symptoms, and body dissatisfaction ‐ and loneliness, also considering interpersonal dependence. Their mediation model identified loneliness as a key mediator between interpersonal dependence and body dissatisfaction for both men and women. Individuals with higher interpersonal dependence appeared more susceptible to loneliness, which in turn contributed to body dissatisfaction. Gender differences emerged, with loneliness significantly related to all ED measures (drive for thinness, bulimic symptoms, and body dissatisfaction) in men but only to body dissatisfaction in women, suggesting a nuanced role of loneliness across genders in ED symptomatology.

In a more recent study, Mason (2024) reported a strong association between loneliness and binge eating, indicating that loneliness not only contributes directly to binge eating behaviours but also moderates the effects of affective symptoms such as depression, anxiety, and body shame, especially in college women. Women experiencing heightened loneliness and emotional distress showed an increased likelihood of engaging in binge eating, pointing to loneliness as an amplifying factor in ED behaviours linked to emotional distress.

Evidence supporting this association is also provided by Marffy et al. (2024), who identified a significant association between loneliness and ED symptom severity, suggesting that emotional isolation may exacerbate ED‐related behaviours. They examined the interaction between loneliness and the frequency of the ‘anorexic voice’ ‐ defined as a person's internal dialogue commenting on weight, shape, eating, and their implications for self‐worth (Pugh et al. 2018) ‐ on the severity of eating disorder symptoms, concluding that both factors independently contribute to symptom severity. Conversely, Hussain (2021) found no significant correlation between loneliness and various dysfunctional eating behaviours. This divergence from other studies suggests that loneliness may play a more intricate role, potentially influenced by additional psychosocial or cultural factors that vary across populations and settings.

Together, these studies underscore loneliness as a complex yet consistent factor in ED behaviours among young adults, with evidence suggesting that loneliness contributes directly and indirectly to ED symptomatology. Despite some discrepancies, the findings generally support the notion that loneliness interacts with emotional and interpersonal dynamics, influencing the severity and persistence of ED behaviours in vulnerable populations.

## 4 Discussion

This systematic review highlights loneliness as a complex, multidimensional construct that interacts with both psychological and physiological processes in ED patients, while also serving as a potential precursor to the disorder's development. Consistent with the structure of the Results section, the discussion is delineated according to clinical and subclinical samples.

### 4.1 Loneliness and ED in Clinical Sample: A Multifaceted Contributor to Pathology

Studies demonstrated that individuals with ED report elevated levels of loneliness, regardless of the specific diagnosis, suggesting that loneliness is a pervasive element across ED pathology rather than confined to particular subtypes such as AN, BN, or BED (Makri et al. 2022). Loneliness is consistently associated with increased severity of ED symptoms (Esplen et al. 2000; Masheb and Grilo 2006; Southward et al. 2014; McNamara et al. 2022; Meneguzzo, Terlizzi, et al. 2024), with studies showing that ED patients report significantly higher loneliness compared to healthy controls (Makri et al. 2022; Coric and Murstein 1993).

Emerging research suggests loneliness functions not only as a consequence of ED but also as both a precipitating factor and a maintaining mechanism in the development and persistence of ED pathology. For instance, Harney et al. (2014) observed reductions in loneliness levels as individuals with ED transitioned from the active illness phase to recovery, highlighting the potential benefits of incorporating loneliness‐targeted interventions into standard ED treatment frameworks. This aligns with the cognitive‐interpersonal maintenance model, which emphasises how loneliness exacerbates social withdrawal and relational difficulties, thereby perpetuating restrictive behaviours characteristic of AN (Treasure and Schmidt 2013).

Furthermore, loneliness is closely associated with the relationship between emotional dysregulation and ED symptoms, particularly in BN e BED (Southward et al. 2014) and appears to intensify emotional vulnerability potentially reinforcing maladaptive coping strategies such as binge eating (Masheb and Grilo 2006). This is further supported by the interpersonal model of binge eating, which suggests that interpersonal difficulties directly generate negative emotions, mediating the link between relational impairments and binge‐eating behaviours. In particular, low affiliative behaviour, characterised by social detachment and emotional distancing, represents a critical interpersonal deficit that amplifies negative affect, thereby exacerbating binge eating and loss‐of‐control behaviours (Ansell et al. 2012; Wilfley et al. 2003). Although the associations between loneliness, emotional dysregulation, and ED symptoms are consistently observed, the cross‐sectional nature of most studies calls for a cautious interpretation of their interrelations. The available data do not support a unidirectional causal pathway; instead, they point towards a reciprocal dynamic, in which emotional dysregulation and ED symptoms may both influence and be influenced by experiences of loneliness. This is consistent with prospective longitudinal evidence highlighting bidirectional relationships between loneliness and disordered eating (Cortés‐García et al. 2022Cortés‐García et al. 2022). Therefore, loneliness may operate not merely as a co‐occurring variable but as an integral component of the emotional and interpersonal context sustaining disordered eating. Integrating interventions that explicitly target loneliness within broader emotion regulation frameworks could therefore strengthen treatment outcomes. Recent research has begun to explore the biological implications of loneliness in individuals with ED. Emerging evidence suggests an association between loneliness, inflammatory markers, and stress‐related biomarkers among ED patients, indicating its potential role in physiological dysregulation (Meneguzzo, Terlizzi, et al. 2024). This aligns with previous findings that chronic loneliness is associated with heightened stress responses and immune dysfunction, factors that can complicate recovery in individuals with ED (Morr et al. 2022). These associations imply that loneliness may exacerbate ED pathology not only through psychological distress but also via physiological pathways, potentially increasing the risk for physical comorbidities. This perspective underscores the need for integrative treatment approaches that address both the psychological and biological dimensions of loneliness in ED patients.

### 4.2 The Role of Loneliness in Subclinical Eating Disorders

A growing body of evidence underscores the significant relationship between loneliness and subclinical ED symptoms, particularly in young adults and college students who engage in disordered eating behaviours and beliefs without meeting full diagnostic criteria (Pritchard and Yalch 2009). Loneliness appears to function as a precursor to more severe ED pathology, emphasising the critical role of early identification and intervention in at‐risk populations (Marffy et al. 2024). In this regard, Wright and Pritchard (2009) demonstrated that loneliness independently predicts disordered eating behaviours, even when controlling for the effects of media exposure, suggesting that loneliness may act as a unique and exacerbating vulnerability factor. Furthermore, prospective longitudinal studies have demonstrated that early experiences of loneliness predict increases in disordered eating behaviours over time, particularly dieting and bulimic behaviours, while conversely, disordered eating can exacerbate subsequent feelings of loneliness, especially in young women (Cortés‐García et al. 2022Cortés‐García et al. 2022).

Psychosocial variables, such as interpersonal dependency, have also been implicated in intensifying loneliness, thereby amplifying body dissatisfaction and other ED symptoms. For instance, Chang et al. (2014) found a strong association between loneliness and bulimic behaviours, potentially related to the inherently isolating nature of these behaviours. Moreover, deficits in social processing, such as difficulties interpreting social signals, increased attention to negative interpersonal cues, and lower affiliative warmth, predispose individuals to loneliness and negative affect, thereby fuelling restrictive and binge eating behaviours (Ansell et al. 2012; Treasure and Schmidt 2013; Wilfley et al. 2003). Consequently, these findings highlight the necessity of incorporating targeted strategies that address interpersonal and emotional needs into preventative interventions for ED. Addressing loneliness as a core element of ED vulnerability could enhance the efficacy of early interventions and reduce the progression towards clinical ED presentations.

Loneliness also interacts with affective symptoms such as depression and anxiety, both of which are prevalent comorbidities in ED populations. Mason (2024) demonstrated that loneliness exacerbates emotional triggers for binge eating, suggesting that loneliness modulates the relationship between affective symptoms and ED behaviours, particularly in at‐risk young adults. While this interplay underscores the relevance of loneliness in shaping ED symptomatology, it likely reflects a broader network of interrelated factors, including depressive symptoms, social anxiety, and early trauma, that may collectively contribute to emotional vulnerability. Recent findings indicate that loneliness may partially mediate the relationship between childhood trauma and ED severity, further highlighting its role within a complex, multifactorial framework (Meneguzzo, Marzotto, et al. 2024). These patterns support a conceptualisation of loneliness not merely as a co‐occurring symptom but as a reinforcing component of the emotional dysregulation underlying ED psychopathology.

Although the number of studies including male participants was limited, the available data suggest that gender may influence how loneliness and social stressors relate to disordered eating. For instance, one clinical study on individuals with BED found that women reported significantly more emotional overeating in response to loneliness than men, highlighting potential gender‐specific affective triggers (Masheb and Grilo 2006). In subclinical samples, other studies have shown that loneliness may mediate disordered eating through different interpersonal vulnerabilities, with female participants generally reporting higher levels of body dissatisfaction and emotional reactivity (Pritchard and Yalch 2009; Wright and Pritchard 2009). Conversely, men may show lower interpersonal trust and reduced self‐disclosure, which can contribute to greater loneliness. This is consistent with findings by Rotenberg et al. (2013), who reported that higher bulimic symptoms were associated with diminished trust, less disclosure, and increased loneliness. Gender differences emerged primarily in symptom severity.

Overall, these findings highlight the need for future research to examine whether distinct social experiences, such as exclusion or ostracism, elicit different emotional and behavioural responses across genders, potentially contributing to the onset or maintenance of disordered eating through divergent psychological mechanisms. Although the current evidence does not allow for definitive conclusions, these aspects should be carefully considered in the development of gender‐sensitive interventions addressing the social and emotional dimensions of eating disorders.

### 4.3 Clinical Implications and Future Directions

The findings of this review indicate a consistent association between loneliness and eating disorder symptomatology across varying levels of clinical severity. It is important to consider the potential overlap between groups classified as clinical and subclinical, particularly given that individuals reporting significant symptoms, although not formally diagnosed, may nonetheless meet diagnostic criteria for conditions such as OSFED as outlined in the DSM‐5‐TR (American Psychiatric Association 2022). At the same time, it is crucial to adopt a balanced perspective regarding loneliness, especially within college‐aged populations. In these groups, certain emotional experiences may reflect typical developmental challenges rather than pathological indicators (Kirwan et al. 2023). Differentiating between normative developmental processes and factors potentially contributing to ED would enhance clinical assessments and support the delivery of more targeted interventions. Despite this nuance, this consideration reinforces the clinical relevance of loneliness across the full spectrum of disordered eating and highlights the need for innovative treatment and prevention strategies. Interventions that integrate emotional and interpersonal dimensions, including loneliness, with behavioural and cognitive components represent a more comprehensive and potentially effective approach to care. Supporting this perspective, growing evidence suggests that loneliness contributes meaningfully to the development and persistence of ED symptoms, particularly by amplifying emotional distress and maladaptive coping mechanisms. Recent evidence from a multi‐centre study reinforces the clinical significance of loneliness in ED, identifying it as a key predictor of health‐related quality of life, independent of BMI and illness duration (Todisco et al. 2024). These findings suggest that loneliness may influence ED severity partly through its impact on overall well‐being.

Incorporating interventions that specifically address loneliness and promote social connectedness could significantly enhance treatment outcomes for ED. Therapies such as Radically Open Dialectical Behaviour Therapy (RO‐DBT) have demonstrated efficacy in treating AN by targeting maladaptive overcontrol or excessive inhibitor control (Lynch 2018; Schroeder et al. 2024) and improving social engagement (Ben‐Porath et al. 2020; Lynch 2018). Adapting these therapies to other ED subtypes provide similar benefits. Similarly, the cognitive‐interpersonal maintenance model of AN emphasises the need for targeted interventions addressing social‐cognitive deficits, emotional recognition, and obsessive‐compulsive traits, given their role in exacerbating social withdrawal and interpersonal difficulties that contribute significantly to chronic loneliness and persistent ED behaviours (Treasure and Schmidt 2013).

For BN and BED, where emotional dysregulation is a prominent feature, interventions that simultaneously address emotion regulation and loneliness may be particularly effective. Standard Dialectical Behaviour Therapy (DBT) has proven successful in enhancing emotion regulation (Ben‐Porath et al. 2020; Chen et al. 2018; Linehan and Chen 2005). Incorporating modules aimed at strengthening social connections could further improve treatment outcomes by addressing underlying vulnerabilities more effectively. Considering the mediating role of loneliness in the relationship between emotional dysregulation and ED symptoms (Southward et al. 2014), a dual‐focus approach targeting both dimensions may provide a more comprehensive intervention strategy. In fact, the interpersonal model of binge eating particularly underscores the importance of addressing specific interpersonal deficits, such as low affiliation and social detachment to reduce negative affect and consequent binge eating behaviours, advocating for the incorporation of targeted interpersonal psychotherapy interventions (Ansell et al. 2012).

Group‐based approaches, including therapy and peer support, should be integrated into treatment plans to strengthen social connections and mitigate loneliness‐related ED behaviours. Integrating social skills training and interpersonal therapy could help patients build healthier relationships and practice interactions in safe environments. These strategies hold promise for reducing loneliness, alleviating body dissatisfaction, and interrupting cycles that sustain ED symptoms.

Digital interventions offer innovative solutions for addressing ED symptoms and loneliness. E‐mental health platforms, online support groups, and teletherapy have been shown to improve access to care and provide meaningful social support for individuals who are hesitant or unable to engage in face‐to‐face therapeutic interventions (Linardon et al. 2020; Sarwar Shah et al. 2019). Virtual reality (VR) technologies further expand these options by simulating social environments, allowing patients to practice social skills and manage anxiety in controlled settings (Gorini et al. 2010). These tools can simultaneously target eating symptoms (Di Natale et al. 2024; Pizzoli et al. 2024; Riva et al. 2023). However, careful implementation is critical to avoid exacerbating social isolation or triggering other adverse effects.

Preventive strategies designed to reduce loneliness could play an essential role in lowering the risk of ED development in subclinical populations, particularly among adolescents and young adults. School‐based initiatives aimed at fostering social inclusion, resilience, and adapting coping mechanisms could prove effective. Additionally, educating parents, teachers, and peers to recognise the signs of loneliness and disordered eating can support early intervention efforts.

Despite the compelling evidence linking loneliness with ED, the existing literature is constrained by notable methodological limitations. Key issues include variability in the measurement of both ED symptoms and loneliness, which limits cross‐study comparability. Although the majority of studies (*n* = 11) employed the UCLA Loneliness Scale, offering a degree of consistency in the operationalisation of loneliness, others used alternative validated instruments, such as the Three‐Item Loneliness Scale and the Friendship Scale. These tools, while conceptually related, assess partially distinct dimensions of loneliness, ranging from general perceptions of social isolation to more affective or relational aspects. This heterogeneity may contribute to variations in the strength and nature of the associations observed between loneliness and ED symptoms. Moreover, none of the studies systematically compared these instruments within the same sample, making it difficult to determine whether specific facets of loneliness are differentially associated with particular ED symptomatology (e.g., binge eating vs. restrictive behaviours). The absence of such comparisons limits interpretability and points to an important direction for future research. Additionally, the predominance of cross‐sectional designs precludes determining causality. This review excluded studies conducted during the COVID‐19 pandemic to reduce confounding related to social restrictions; however, this decision may have led to the omission of relevant data on how acute social stress influences the relationship between loneliness and ED symptoms. Moreover, most of the research considered is conducted within Western contexts, raising concerns about cultural generalisability. Since loneliness differs between collectivistic and individualistic societies (Lykes and Kemmelmeier 2014), exploring how cultural norms and community structures influence loneliness and ED risk could be crucial. This review included one study from Pakistan (Hussain 2021), uniquely reporting no relationship between loneliness and dysfunctional eating patterns, further highlighting cultural variability. Future research should prioritise longitudinal designs involving underrepresented populations to clarify cross‐cultural variability and address existing inconsistencies, such as gender differences. Integrating biological, mechanistic, and real‐time assessment methods, including Ecological Momentary Assessment (EMA), can help capture temporal dynamics of loneliness and ED behaviours, providing insight into potential causal pathways and fluctuations. Furthermore, examining individual differences like social reward sensitivity and attachment style could clarify the conditions under which loneliness increases disordered eating risk, ultimately facilitating the development of culturally sensitive and individually tailored interventions. Given the complex relationship between ED and loneliness, a biopsychosocial approach is crucial. Effective interventions should address individual psychological factors alongside broader social and environmental contexts. Community‐based programs that promote social engagement and reduce stigma can support recovery. Moreover, combining technological innovations with human‐centred strategies may revolutionise treatment. For instance, VR‐enhanced social skills training and interventions powered by Artificial Intelligence could significantly improve therapeutic outcomes.

## 5 Conclusion

In conclusion, loneliness emerges as a transdiagnostic factor with significant psychological and biological relevance to ED psychopathology. It plays a critical role in emotional dysregulation, intensifies affective symptoms, and is associated with physiological stress responses that perpetuate ED. These findings align with prior research emphasising loneliness as core psychosocial determinant in mental health disorders (Hawkley and Cacioppo 2010; Levine 2012; Mann et al. 2022) and clarifying its complex interactions with emotional dysregulation and social isolation in sustaining ED pathology (Dingemans et al. 2017; Leigh‐Hunt et al. 2017).

This review highlights the critical need to incorporate interventions targeting loneliness into ED treatment protocols. Specifically, strategies that integrate social support and emotional regulation training could address this underlying factor more effectively. Adopting innovative approaches, such as the use of advanced technologies and community‐based programs, could extend the accessibility and efficacy of these interventions.

Emphasising the social aspects of ED within research and clinical practice represents a promising opportunity to improve patient outcomes. Prioritising the prevention of loneliness in contemporary society is critical, as it plays a fundamental role in mitigating vulnerability to psychological disorders, including ED while promoting overall mental health.

## Author Contributions

**Elisa Rabarbari**: conceptualisation, data curation, formal analysis, investigation, methodology, writing – original draft, writing – review and editing. **Chiara Rossi**: data curation, formal analysis, investigation. methodology, writing – review and editing. **Fabio Frisone**: data curation, formal analysis, investigation. methodology, writing – review and editing. **Giuseppe Riva**: project administration, funding acquisition, supervision, writing – review and editing.

## Conflicts of Interest

The authors declare no conflicts of interest.

## Data Availability

The data that support the findings of this systematic review were derived from previously published studies available in the public domain. All sources are listed in the reference section of the manuscript.
